# Gait analysis under the lens of statistical physics

**DOI:** 10.1016/j.csbj.2022.06.022

**Published:** 2022-06-18

**Authors:** Massimiliano Zanin, Felipe Olivares, Irene Pulido-Valdeolivas, Estrella Rausell, David Gomez-Andres

**Affiliations:** a Instituto de Física Interdisciplinar y Sistemas Complejos IFISC (CSIC-UIB), Campus UIB, Palma de Mallorca 07122, Spain; b Department of Anatomy, Histology and Neuroscience, School of Medicine, Universidad Autónoma de Madrid, Calle del Arzobispo Morcillo 2, Madrid 28029, Spain; c Pediatric Neurology, Vall d’Hebron Institut de Recerca (VHIR), Hospital Universitari Vall d’Hebron, Vall d’Hebron Barcelona Hospital Campus, ERN-RND & EURO-NMD, Pg. de la Vall d’Hebron 119-129, Barcelona 08035, Spain

**Keywords:** Human gait, Entropy, Maximum Lyapunov exponent, Multi-fractal analysis, Irreversibility

## Abstract

Human gait is a fundamental activity, essential for the survival of the individual, and an emergent property of the interactions between complex physical and cognitive processes. Gait is altered in many situations, due both to external constraints, as e.g. paced walk, and to physical and neurological pathologies. Its study is therefore important as a way of improving the quality of life of patients, but also as a door to understanding the inner working of the human nervous system. In this review we explore how four statistical physics concepts have been used to characterise normal and pathological gait: entropy, maximum Lyapunov exponent, multi-fractal analysis and irreversibility. Beyond some basic definitions, we present the main results that have been obtained in this field, as well as a discussion of the main limitations researchers have dealt and will have to deal with. We finally conclude with some biomedical considerations and avenues for further development.

## Graphical abstract

**Figure:**
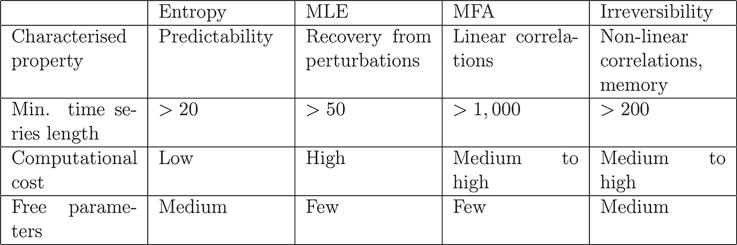

## 1 Introduction

Walking is the main way humans use for independent self-translation around the world. Normal human walking is a method of locomotion involving the use of the two legs, alternately, to provide both support and propulsion with at least one foot being in contact with the ground at all times [1]. Gait is a very individual trait in healthy subjects that can even be used for personal identification [2], but it changes with age [3], [4] and can be modified by emotions [5], exercise-related or cognitive fatigue [6], or environmental factors [7]. Walking is formed by a sequence of gait cycles, its basic and fundamental unit. These can be defined as the combination of motor phenomena that occur between two floor contacts (usually heel contacts) of the same foot, and is composed of two phases: 1) the stance one, in which the foot maintain contact with the ground; and 2) the swing one, in which the limb moves forward in a sort of pendular motion [1]. Walking then emerges from interleaving left and right gait cycles.

Bipedal walking is one of the main milestones in hominid evolution [8], and independent walking is the most relevant milestone in motor development during infancy [9]. This is because walking requires an extremely well coordinated, finely modulated activation of muscles by the central nervous system [10], [11]. In contrast to a simplistic idea of quasi-automatic movements produced by spinal circuits, walking is an emergent property of the central nervous system; and, as any voluntary movement, it is determined by an intense neural control involving a complex network formed by cortical regions, basal ganglia, brainstem centres and spinal cord circuits [12].

In the field of basic neuroscience, walking has received significant attention as a model of human movement, due both to its functional relevance, and to the advantage of being a cyclic movement that can be measured and treated as a complex signal [13]. Similar attention can be found in medicine due to the impairment resulting from multiple disorders, such as highly prevalent musculo-skeletal diseases or devastating neurological disorders [14]. The analysis of gait patterns can tell us significant information about these disorders and help us in therapeutic decision making [15].

Many neurological and neurodegenerative diseases have a definitive effect on motor control. Subsequent movement impairments, in particular gait abnormalities, are very frequent, altering daily life and causing dependency. In normal healthy conditions, the coordination of joint movements during gait is tightly controlled by neural motor brain structures. As a whole, these brain structures regulate many factors contributing to the appropriate sequence of flexor–extensor muscle contractions, the final result being a precise movement of the joint chains and the translation of the body’s center of gravity in bipedal posture [16]. Lesions in any of the various components of the cerebral motor system (including motor cortices, basal ganglia, thalamus, cerebellum, corticospinal pathways, and ascending somatosensory pathways) alter the output signals of the postsynaptic motor neuronal spinal cord circuitry and, therefore, the sequence of muscle contraction [17]. Gait motor performance is hence altered at first instance, impeding the correct translation of the body. However, gait is a cognitive propositive action of high importance for the brain, and the whole nervous system adapts its dynamics to achieve the target. Several plastic mechanisms are then triggered, including, for instance, the re-arrangement of synaptic connections [17]. This ultimately results in the generation of newly-configured sequences of muscle contractions, executing a sort of “maladaptive” gait. While the cognitive target is achieved, the movement might not properly be adjusted to the biomechanical properties of the joint’s soft tissues, muscles and bones and to cellular metabolic needs, thus yielding torque-related deformities. Those newly-generated signals are a reflection of brain adaptation, and the quantitative evaluation of the differences between the neurological patients’ and the control subjects’ signals is essential for the interpretation of the prognosis of the disease and for the design of personalised therapies. Studying and understanding human gait is thus of major importance, both for guiding medical interventions and as a door for shedding light on the functioning of the nervous system. The advent of new technologies has made possible the quantification of the different motor phenomena involved in gait pattern configuration [18], and different mathematical tools have been applied to these data [19], [20].

Within this large field, the application of statistical physics concepts has been receiving an increasing attention. It is not difficult to identify the reasons behind this surge. One of the main objectives of this branch of physics is the characterisation of the constituents of a system (i.e. the micro-scale), when only the emergent global dynamics (i.e. the macro-scale) is accessible and thus observable [21]. Making a parallelism with genetics, this is equivalent to trying to characterise the genotype, when only the phenotype of an organism (and changes thereof) can be observed. The same problem is found in gait, which is the result of a chain of interactions starting from the central nervous system and ending in the peripheral tissues; yet, only the final output, and not the intermediate steps, are easily observable. Among the many metrics that have been developed within statistical physics to this end, a few of them stands out for describing concepts well aligned with neuroscience: to illustrate, entropy is related to the predictability of a signal, and the lack of predictability can easily be associated to a lack of control by the lesioned brain; and the maximum Lyapunov exponent describes the response of the system to perturbations, which translates to the feedback control mechanisms acting during gait. On top of this, most statistical physics metrics are easy to compute, and are built on solid theoretical foundations.

In this review, we aim at creating a stronger bridge between clinical gait analysis and statistical physics, and at fostering the flow of ideas from the latter to the former. For that, physicists have to understand the idiosyncrasies and problems behind gait data; and, at the same time, physicians need to better understand what is assessed by statistical physics metrics, which at times can be quite abstract. We focuson how four fundamental statistical physics concepts have been applied to instrumental gait analysis, namely: entropy, maximum Lyapunov exponent, multi-fractal analysis and irreversibility. These have been chosen for being representative of basic statistical physics concepts (including predictability, fluctuations and time asymmetry); for being computationally affordable; and for having widely been used in gait analysis. We discuss how these concepts have been applied to different types of gait data, and the main results obtained in different pathologies. We finally close this review with a discussion of the limitations of the methods hitherto proposed, of some conceptual mistakes we have found in the literature, and of avenues for further development.

## 2 Basics of instrumented gait analysis

People has been interested in analysing walking since the earliest moments of science. Aristotle (384–322 BCE) is the author of the earliest recorded comments and theories on the movement of humans and animals [22]. Progress was afterwards made through the experiments and theories of Giovanni Borelli (1608–1679), on tendon biomechanics, and of Willhelm (1804–1891) and Eduard (1806–1871) Weber, on the anatomy and biomechanics of walking. Other notable contributors include Jules Etienne Marey (1830–1904), Eadweard Muybridge (1830–1904), and Gaston Carlet (1849–1892), who developed a shoe with three pressure transducers built into the sole; this was the first recording of the double bump of the ground reaction force vector [23]. The major developments in the early twentieth century were force plates and the understanding of kinetics [22]. It was not until the advent of modern computers that clinical gait analysis became widely available. Instrumented (or computerised) gait analysis (IGA) is now a complex discipline based on powerful systems of measurement with different degrees of complexity, strong neuro-scientific research and widely spread clinical application. Still, IGA has not reached its maximal technology capacity yet. In the years to come, better and cheaper systems may be incorporated to clinical care and will provide clinicians with a large quantity of precise information of motor performance, which needs to be processed and presented in a useful way.

When it comes to the numerical analysis of IGA data, three aspects have to be clarified: the type of data; how these data are represented; and under which conditions they are recorded.

Regarding the first aspect, motor phenomena can be evaluated by means of different recordings - see Table 1. Spatiotemporal parameters are those that assess how the body moves as a whole (e.g. speed) or how the gait cycle is configured (e.g. stance time). Kinematic parameters assess how the displacement of particular body segments around the different joints is made in space (e.g. knee flexion). Kinetic parameters assess the forces that provoke the displacement of the body or particular body segments (e.g. power, torque), measured through force plates. By means of models, we can extract torques and powers for the different joints, and even for particular muscles. The muscle activity along gait cycle can be assessed by superficial electromyographic recordings. Finally, the metabolic cost of walking can be measured indirectly by calorimetry and metabolic cost measurements (e.g. CO_2_).

**Table 1:** Different types of parameters in instrumented gait analysis.

	Question	Definition	Example
Spatiotemporal parameters	What?	Motion of the whole body	Walking speed, step cadence
Kinematic parameters	What?	Motion of individual joints	Knee flexion
Kinetic parameters	How?	Forces, work and power behind the motion	Knee flexor moment
Electromyographic parameters	Why?	EMG activation of individual muscles	Activation of the vastus lateralis
Individual muscle information	Why?	Data of muscle performance from inverse dynamic modelling	Force produced by vastus lateralis
Oxygen/ CO_2_ consumption data	How much?	Energy consumption	Oxygen consumption during walking

Data from IGA can be analysed as scalar values, giving information of a particular feature in a precise moment of the cycle (e.g. knee flexion at initial contact), or averaged along the whole cycle (e.g. range of knee flexion). Alternatively, they can be analysed as vectors or matrices by multivariate statistical and machine-learning approaches [19], [20], [24] - the former being the focus of this review.

Finally, gait can be analysed in different environments. Traditional IGA uses laboratories in which highly precise systems quantify different aspects of human movement, for instance on instrumented aisles or treadmills. Additionally, virtual reality systems have been incorporated, allowing the integration of non-portable equipment into real environments [25]. Finally, systems like instrumented insoles, accelerometers, or even mobile phones, have allowed the assessment of gait in natural environments [26].

## 3 Entropy

Entropy, in its physical interpretation of the quantity of disorder, randomness, or uncertainty about the dynamics of a system, has a long history of applications in biology and medicine [27], [28]. When applied to IGA, it allows to assess how repetitive and controlled the gait cycles are. As a lesioned system and its subsequent adaptive mechanisms can only provide a restricted number of possible movement patterns, and they can only enforce them through a weaker control, a reduction is to be expected in the entropy of a number of cinematic parameters. To the best of our knowledge, the first application of entropy measures to gait time series was proposed in 2003 [29], comparing healthy subjects walking in different conditions (spontaneous walking vs. walking paced by a metronome). Since then, the number of studies has exploded; not just in the number of conditions, but also in the number of applied entropy metrics. For the sake of completeness, the most important are sketched below.

• Shannon and symbolic entropies. Shannon’s original formulation of the entropy [30] is calculated over a continuous probability distribution f(x) as: $S[f(x)]=-\int f(x)\log_{2}f(x)\mathrm{dx}$. When the base of the logarithm is set to 2, it represents the information needed, in terms of number of bits, to describe the distribution. When continuous values are not available, and in general when it is convenient to discretise the signal to reduce noise and artefacts [31], the resulting entropy is known as symbolic [32].
• Approximate (ApEn) and Sample (SampEn) entropy. Building on the concept of Kolmogorov-Sinai entropy, ApEn measures the predictability of time series by assessing how probable are two sub-windows of it, which are similar between themselves, of also evolving in a similar manner [33]. The greater is this probability, the more regular (or predictable) is the time series, and hence the smaller the resulting ApEn value. SampEn is a further modification of the same idea, in which the comparison of a sub-window with itself is not taken into account; this reduces the bias of the metric, at the cost of not being a true information measure anymore [34]. More comprehensive comparisons of both metrics can be found in Refs. [35], [36].
• Permutation entropy. Metric representing the information encoded in the permutation patterns associated to a time series, that is, in the order relations among consecutive values [37], [38], [39]. It is assessed by dividing the time series into short windows; by calculating the corresponding permutation pattern, i.e. the order required to sort the elements of such windows in ascending order; and finally by calculating the Shannon entropy of the resulting pattern probability distribution.
• Multiscale entropy. Previously described entropy metrics are calculated over all values available in the time series, thus inherently analysing the time scale induced by the temporal resolution of data. Healthy (and pathological) physiologic dynamics nevertheless develop over a multitude of scales, whose heterogeneity can be described by calculating a multiscale entropy. As originally proposed [40], [41], this involves two steps: firstly, the reconstruction of consecutive coarse-grained time series $\left\{y^{\tau }\right\}$, such that $y^{\tau }(t)=\frac{1}{\tau }\sum_{i=(j-1)\tau +1}^{j\tau }x(i)$; in other words, the time series is splitted in non-overlapping sub-windows of size τ, which are represented by the corresponding average value. Secondly, any suitable entropy metric is calculated over the new time series $y^{\tau }(t)$, for then obtaining the evolution of the entropy as a function of τ. Many alternatives to this initial approach have been subsequently proposed, see for instance [42]. It can be appreciated that, more than to a specific metric, multiscale refers to a way of pre-processing the data; as such, this approach can be found in conjunction with SampEn [29], [43], [44], [45], [46], [47], symbolic entropy [48], [49], [50], or approximate entropy [51].
• Control entropy. One assumption underlying all previously described entropy measures is that the analysed time series are stationary in a statistical sense, something not usually holding in biology and medicine. In order to tackle this issue, the concept of control entropy was introduced, as the entropy of the differences between neighbouring values, as opposed to the values themselves [52]. Mathematically, given a time series x(t), this can be calculated as: $\mathrm{CE}[x(t)](t)=\mathrm{SampEn}[\delta (\frac{\mathrm{dx}}{\mathrm{dt}}(t,t+w))]$, where δ and *w* are the parameters used to calculate the SampEn as described above.

Additional alternatives of entropy metrics, not reviewed here for the sake of conciseness, include the analysis of multi-scale entropy through Discrete Wavelet Transform [53]; quaternion entropy [54]; quantized dynamical entropy [55], [56], [57]; diffusion entropy, i.e. the entropy of the diffusion process generated by a time series [58], [59]; intrinsic mode entropy [60], [61]; differential entropy [62]; cross entropy [63], [64]; and persistent entropy [65], [66]. The precision and relevance of some of these metrics have directly been compared, as for instance in Refs. [48], [61], [67], [68], [50]; and analyses have been performed on the importance of different parameters [35], [69], [70], [67]. There are nevertheless no guidelines about which metric is better for the analysis of a given system, leaving the doors open to multiple options.

The analysis of gait entropy should logically start with healthy subjects, as a way of shedding light on the mechanisms behind the normal control performed by the central nervous system; many studies have therefore focused on the effects of different walking speeds, either spontaneous, forced (through a metronome) or by means of slopes [71]. The influence of walking speed on the different explored metrics for entropy in IGA is complex. Higher walking speeds seem to be related with an increased entropy in kinematic time series [72], [54], reflecting that the SNC might be either relaxing its otherwise tight control when increasing velocity to adapt to more spurious unexpected interferences, or because there is no time to imprint the same tight control as when walking slow. On the other hand, the entropy of the time of stride seems to evolve in the opposite direction, showing increased entropy with lower walking speeds [73], [74]. Another study further showed no statistically significance difference for all paced walking speeds [29]. Such complexity may be the results of both the small differences induced by the walking speed, and of the use of different entropy metrics. It is finally worth noting two additional studies on healthy subjects, specifically runners [75] and toddlers [43].

Within the analysis of healthy individuals, a special place should be reserved to studies devoted to the analysis of gait of elderly people. The aim is usually to forecast, and eventually to understand the reasons behind, the risk of falling, as this is associated to major comorbidities and healthcare costs [76], [77], [78]. Obtained results are somewhat contradictory. Specifically, most of the works generally found an increased entropy in elderly individuals [79], [80], [45], [57], [81]. Moreover, this increase seems related with falls. This suggests that elders have lost their adaptive capacity due to the natural deterioration of the brain, with movement being less regulated, specifically the transmission of the somatosensory feedback to cerebellum that modulates the response to interferences of the external world to gait is altered, as well as the continuous control of antigravitation muscles by brainstem mechanisms like reticulospinal or vestibulospinal systems [82]; but some found statistically not significant differences [44], [83]. Finally, Ref. [84] found a complex relationship with the minimum toe clearance (expression of somatosensory feedback deficit of joint positions to motor control in the swing phase), with elders usually having higher entropy, reflecting that the motor response is not regulated because of the impoverishment of somatosensory inputs; but a lower entropy when large values of the clearance are considered - an effect more marked when considering the falls risk group. It is also worth noting Ref. [64], which proposed a clinical classification model reaching an accuracy of 90%.

When moving to the analysis of the gait of patients suffering from specific diseases, a group of three stands out for being usually tackled together. These include Parkinson’s Disease [85], [53], [86], [87], [88], [51], [46], [89], [90], [47], [66], Huntington’s disease [85], [53], [86], [91], [51], [46], [90], and amyotrophic lateral sclerosis [85], [53], [86], [51]. Note that, in spite of the similar results they yield, these pathologies are substantially different, the first two being related to the basal ganglia, which control the fine processing of movements temporally regulating the eccentric/concentric muscle contraction; and the latter one to the motor neurons of the cerebral cortex, brainstem and spinal cord, which control the concentric contraction of muscles and thus the force, power and joint torque. One would expect that after a lesion of motor neurons, the subsequent problems in agonist/antagonist concentric contraction and spasticity would initially decrease the entropy, and that it would remain lower while the system is compensating and fixing a new configured kinematic pattern that allows locomotion. Then, after larger lesions are produced (due to the degenerative nature of these pathologies), entropy should increase as a reflection of the loss of adaptive capability of finding a functional gait parameter configuration to walk. On the other hand, basal ganglia lesions should disrupt the fine control of movement, resulting in an increase of entropy due to a lack of adaptive capacity. These changes may vary, influenced both by the thresholds and scales in the entropy metrics. Most analyses indicate that patients suffering from these three diseases display higher entropy [89], [90], [47]; the only exception can be found in Ref. [51], where few significant differences were found between the amyotrophic lateral sclerosis (ALS) disease and control groups, and Ref. [85], where the entropy of the control group was higher. It is unfortunately difficult to understand where these differences come from, due to the multiple available entropy metrics, and to the different ways data have been pre-processed. In spite of this, having comparable data for these three diseases together has allowed the creation of classification models for predicting the specific condition of each patient, reaching accuracies of 85% [86], 91% [90], 98% [66], and above 99% [46].

Other diseases that have been studied in the literature include symptomatic knee osteoarthritis [92]; diabetic foot [93]; lumbar spinal stenosis [62]; hemiparetic patients following stroke [94], [95]; multiple sclerosis [96]; Down syndrome [97]; cerebral palsy [98]; Alzheimer’s Disease [99]; and peripheral arterial disease [100]. In almost all cases, a lower entropy has been detected in the gait of patients, when compared to control subjects [97], [92], [96], [93]; the only exception is a higher entropy for ankle, knee, and hip in patients with peripheral arterial disease [100]. Additionally, entropy metrics have been proposed as features for training automatic classification models [62], [95].

## 4 Maximum Lyapunov exponent

The maximum Lyapunov exponent (MLE) is based on the Lyapunov’s theory of dynamic stability, initially formulated to assess the sensitivity of a mechanical system to small perturbations [101], [102]. In short, given a dynamical system, it is based on calculating the evolution of two trajectories starting from very near points, and in assessing how much these trajectories diverge with time. Thus, large values of the MLE imply that small perturbation can result in a very different evolution, and a lack of control on the evolution of the system; the application to gait is thus only natural [103].

From an algorithmic point of view, two main ways have been proposed to estimate the MLE: the algorithm of Wolf et al. [104] (also called the W-algorithm), and the algorithm of Rosenstein et al. [105] (the R-algorithm). While the latter is in principle more suitable for the analysis of short time series, the differences between both can be more complex [106]. Additionally, the computation of these metrics can be performed on a single or multiple strides, called respectively short- and long-term MLE; while the former is more clearly related to the structure of the gait, and especially on the probability of falls, the latter has been found to yield complementary information [107]. Any result should therefore be interpreted with caution.

As expected, the maximum Lyapunov exponent has firstly been estimated in healthy subjects. Beyond several studies addressing the problems associated with its estimation [108], [109], [110], [61], it is worth noting a study showcasing the use of this metric, by comparing the temporal variability between walking in unstable shoes and walking in a normal athletic-type control shoe [111]. Results indicate a higher maximum Lyapunov exponent when walking in unstable shoes, possibly reflecting the larger variability in walking patterns by them forced.

Several pathologies have also been studied, the most important being ageing [112], [108], [113], [109]; correlations have been found between the capacity of maintaining dynamical stability and age, but also with strength and amount of physical activity. Other examples of conditions include claudication [107], cerebella damage [114], models of high falling risk [110], Down’s syndrome [97], multiple sclerosis [115], peripheral arterial disease [100], reconstruction of the anterior cruciate ligament [116], and knee osteoarthritis [117]. In all of these, external interferences exist, including the noises induced by peripheral lesions of soft or hard tissues, like muscle, ligaments, bones or joint tissues, which introduce disturbances in the final response of the motor command and in the somatosensory feedback to the motor system [82]. This would promote modifications in the output signals. A metric also based on the analysis of the trajectory followed by a dynamical system, and thus related to the maximum Lyapunov exponent, is the correlation dimension [118], [119], [120]. Roughly speaking, it represents the probability of two arbitrary points to be closer than a given distance, as a function of such distance. While less attention has been devoted to this concept in the context of gait analysis, it is worth noting a few works that have applied this metric, usually in conjunction with the MLE. These include the analysis of healthy individuals [121], [122], as well as patients suffering from osteoarthritis [123], [124] and neurodegenerative diseases [125], [126].

As a final note, it is worth considering Ref. [127]; beyond studying healthy subjects gait, it also offers a free software for Window® environment to compute several dynamical metrics, both on gait and other types of time series.

## 5 Multi-fractal analysis

Long-range correlations and fractal-like properties are present in a wide range of natural phenomena [128], [129]. The Hurst exponent is one of the most reliable parameters to quantify the scaling law for these temporal correlations, specially when dealing with real-world time series [130], [131]. In the simplest case, only one scaling index is necessary to characterise the global linear correlations in a sequence, namely, mono-fractal. However, when the scaling is not a global but a local property, one scaling exponent is not sufficient to unveil the interplay between a superposition of subsets, each characterised by a different scaling exponent, leading to what is known as multi-fractality [132]. It is important to point out that multi-scaling can be originated by the presence of non-linear correlations and heavy-tail distributions [133].

The most used methodology for estimating scaling exponents is the so-called Multifractal Detrended Fluctuations Analysis (MFDFA) [133]. Briefly, it studies the fluctuations on data sequences by systematically eliminating the *m*th-degree polynomial trends over windows of different sizes *s*. Then, the *q*-Generalised Fluctuation Function $F_{q}(s,m)$ is estimated, defined as the averaged variance of the detrended time series and weighted to a factor *q*. For long-term correlated data, $F_{q}(s,m)$ scales as $s^{h(q)}$ inside a certain range of *s*. For more details about the algorithm, see [133]. For q=2, the classical Hurst exponent is retrieved h(2)=H, (0<H<1), that quantifies the linear correlations in the sequence. H=1/2 stands for memory-less fluctuations. Persistence, i.e. positive memory, is related to H>1/2, while negative memory or anti-persistent correlations correspond to H<1/2. For mono-fractal data, *h* is independent of *q* and equal to *H*, since only one scaling exponent is enough; on the other hand, *h* decreasing with *q* is a result of a multi-fractal nature, which can be described through a set of scaling parameters $\left\{h_{q}\right\}$. It has to be noted that other approaches are also sometimes used to study multi-fractal properties, including those based on wavelet transforms [134], [135], and improved methods based on Fractal Analysis [136], [137]. We start reviewing the application of this concept to gait data by initially focusing on long-range linear correlations, as measured by the Hurst exponent *H*, as these were historically the first to be considered. Primarily, long-range fractal-like correlations (extended over hundred of steps) were found in stride interval fluctuations from healthy young people [138], [139]. One year later, it was shown that these correlations are stable up to thousand of strides at three walking rates (usual or self-selected, slow and fast) [140]. Additionally, Hausdorff and co-workers showed that during metronomic walking, the stride interval becomes uncorrelated independently of the walking rate [140]. Opposed to this result, Delignieres et al. [141] found anti-persistent fluctuations in metronomically constrained walking, and that slow walking can be considered an anti-persistent non-stationary walk rather than a strongly persistent noise. Subsequent studies found that long-range correlations are also present when healthy subjects run [142], [143], or when treadmill locomotion is imposed [144], although correlations are reduced. These results provide evidence that long-range temporal correlations exist at a wide range of gait speeds in healthy young adults. Moreover, models are able to generate sequences reproducing the experimental long-range temporal correlations [145], [146].

Further studies showed that long-range correlations are reduced with maturation [147] and in elderly healthy subjects [148], since there is age-related decline in the general chemical and electrical activity of the micro and macro circuitries of the basal ganglia, which affects mobility, imposing deconfigured neural motor commands that induce longer lasting movements. Besides, physiological ageing brings a decrease in muscle mass and strength [149], [150], joint somatosensory inputs (conscient proprioception) might also be decreased [151], [152], and there is a higher probability of suffering arthrosis and joint deformity [153]. All those factors might also reduce the longe range correlation of movements. On the other hand, Malatesta et al. [154] found no significant differences in the temporal correlations between healthy young, 65-yr-old and octogenarians subjects walking on a treadmill, which makes sense if we take into account that the treadmill is imposing its own rhythm as a pacemaker. An additional study by Herman and co-workers proposed to characterise the “cautious” gait of the elderly [155]. They considered subjects with High Level of Gait Disorder (HLGD, i.e. walking difficulty not attributable to a chronic condition or disability), and found that long-term correlations are significantly lower in fallers compared with non-fallers [155], suggesting that a decrease in the correlations is an indicator of fear of walking.

Pathological alterations on the motor system also induce a decrease in the long-range correlations in the stride interval fluctuations. Such correlations were lower compared with controls when subjects suffer from Huntington [148] and Parkinson’s diseases [156], [144], [157], both pathologies affecting the basal ganglia, whose disability in coordinating the appropriate sequence of muscle contraction introduces tremor or discoordinated movements that increase short range noise. However, stride interval fluctuations from subjects with advanced amyotrophic lateral sclerosis also showed lower degree of long-range correlations, as one would expect from a completely deconfigured neural command that hinder movement, although for this disease the basal ganglia is intact and does not generate short range noise. It was additionally found that treadmill walking reduced the scaling exponent in healthy controls but not in subjects with Parkinson’s disease [144]. On the other hand, the use of medication in subjects suffering of Parkinson’s can be identified when rest tremors are studied, through an increase in the correlations in the velocity signals [53], [95]. Surprisingly, peripheral neuropathy does not alter the temporal correlations of stride intervals of gait, despite these patients tending to walk slowly [158]. Withinn the general context, these findings contribute to the idea that changes in the scaling laws, i.e. the reduction in the correlations, are largely a reflection of a deterioration of the central control of gait, and not simply a reflection of a slower walking speed [158].

Beyond the detection of long-range linear correlations, multi-fractal analysis allows assessing the presence of non-linear ones, which are described by the scaling parameters $\left\{h_{q}\right\}$. Note that the presence of non-linear correlations, multi-fractal scalings, or multi-fractal properties, are all synonyms and are here used interchangeably. Multi-fractal scaling was primarily observed from theoretical models, conjecturing a slightly multi-fractal fluctuations in several gait regimens [146] and its decrease with maturation [145]. Subsequently weak multi-scaling was experimentally confirmed in both free and metronomically triggered conditions in healthy subjects [135], [159], [160], being the unconstrained slow and fast paces the most multi-fractal, and the former one actually being an anti-persistent walk, possibly due to a non-stationarity generated by a loss of concentration while trying to follow the pace. Weak multi-fractality has also been reported by Ivanov et al. [161]. Most recently, Dutta et al. [162] experimentally showed that stride intervals from both healthy subjects and patients with neurodegenerative diseases (Huntington’s and Parkinson’s) present multi-fractal properties, as well as from subjects suffering amyotrophic lateral sclerosis [163], with healthy subjects having higher degree of multi-fractality compared to patients. As opposed to these results, in [164], [165] it was reported that, compared to healthy controls, multi-fractality is higher in individuals with the aforementioned diseases. Further, they found a wider degree of multi-scaling in both children and healthy older adults. On the other hand, Ducharme and co-workers [166], [167] found that healthy subjects generate mono-fractal stride-to-stride intervals for unperturbed walking, and multi-fractal when perturbed. This opposing multi-scaling feature is probably due to a different data acquisition procedure [167]. Moreover, multi-fractality has been also observed in the walking of patients with Parkinson’s during a keystroke [168], and in velocity signals of rest tremors [53], [95]. In most cases, the main source of the multi-scaling is the presence of long-range correlations, rather than the distribution. Remarkably, by comparing the multi-scaling of the correlations between the two feet of a patient, a discrimination between Huntington’s and Parkinson’s diseases is achieved [169]; and such correlation quantifies the degree of the neurodegenerative pathology [170]. Lastly, Ihlen and Vereijken [171] proposed to identify multi-scaling in human gait by analysing the interplay between local temporal correlations and local magnitude of the stride time variability. It is finally worth mentioning that a detailed analysis on the optimisation of the parameters of the fractal approach used in order to obtain reliable results for finite-size measurements can be found in [172].

## 6 Irreversibility

Time irreversibility is formally defined as the lack of invariance of the statistical properties of a system (or a time series) under the operation of time reversal; more intuitively, it can be described as whether a time series can or cannot be recognised from its time-reversed version. To illustrate, imagine watching a movie of an ideal pendulum: it would not be possible to decide whether the movie is played forward or backward, as both would be identical (except for a change in the sign of the velocity). On the other hand, the classical example of an irreversible movie is the one depicting ice cubes melting in a glass; its time-reversed version, with water solidifying in regular structures, is unnatural at best. Time irreversibility is a fundamental property of non-equilibrium systems, and stems from two properties observed in many real-world systems: the presence of non-conservative forces, i.e. of memory [173], [174], and of non-linear dynamics [175]. It is not surprising that irreversibility metrics and tests have been applied to many medical problems, including the characterisation of Parkinson’s disease tremors [176]; of brain dynamics through corresponding electroencephalographic (EEG) recordings [177], [178], [179], [180]; and of cardiac dynamics in different conditions [181], [182], [183]. Nevertheless, and as opposed to what seen for other metrics, irreversibility has mostly been neglected by the gait community - some reasons for this will be discussed in Section 7.

It is intuitive that gait should be an irreversible dynamics, as brain signals to the muscles that provoke joint movement must be continuous and coordinated; in other words, they are the result of a computation in which memory (the past position and movements of the body) plays an important part. This idea was firstly used in 2003 by Ref. [184], and later by Ref. [185] along similar lines; this was nevertheless not numerically checked, and was only used to justify the application of Left-to-Right Hidden Markov Models. More recently, two papers explored the use of irreversibility as marker of pathology. Firstly, Ref. [186] analysed time series of patients with peripheral arterial disease; a statistically significant lower irreversibility was found in patients on the Y axis of both legs with respect to the control group, reflecting a reduced repertoire of possible responses to an otherwise healthy normal neural command. The next year, Ref. [99] analysed gait kinematic time series for patients with mild cognitive decline and early Alzheimer’s dementia; a more complex scenario was depicted, with some joint movements displaying an increased irreversibility (e.g. ankle rotation in mild cognitive decline patients, reflecting an adaptation to dynamically correct the ankle to increase the base of support [187]), while others a marked decrease (e.g. pelvic tilt in both pathologies, which tends to be fixed in a mid flexo/extension position with lower movement range, in order to increase stability during load transfer).

## 7 Conclusions and challenges ahead

Instrumented gait analysis (IGA) is steadily increasing in importance, both for understanding the inner mechanisms of brain computation, and the effects that different pathologies have on this essential aspect of everyday’s life. IGA is nevertheless only as useful as the metrics extracted therefrom, hence the importance of defining and assessing suitable quantifiers. In this contribution we have reviewed how four statistical physics concepts, namely entropy, maximum Lyapunov exponent, multi-fractal analysis, and irreversibility, have been used to characterise human gait, both in health and pathologies. While coming from the same scientific field, these four concepts are substantially different, both in terms of the property of the system they assess, and of the requirements on the analysed data. A synthesis of these differences is proposed in Table 2. It has nevertheless to be taken into account that this is a simplification, as for instance different algorithms, e.g. for estimating entropy, have different requirements in terms of time series length, and also have different numbers of parameters that have to be tuned. Additionally, Table 3 reports a synthesis of the obtained results for five major pathologies, organised in main trends and exceptions for each metric, and further reporting the gait data used in the analysis.

**Table 2:** Main characteristics of the metrics considered in this study; see main text for details.

	Entropy	MLE	MFA	Irreversibility
Characterised property	Predictability	Recovery from perturbations	Linear/non-linear correlations	Computation, memory
Min. time series length	>20	>50	>1,000	>200
Computational cost	Low	High	Medium to high	Medium to high
Free parameters	Medium	Few	Few	Medium

**Table 3:** Synthesis of the main results observed in the Literature for five major pathological conditions. Acronyms in italic and superscript indicate the type of data analysed by each work. jap: joint angles and positions; ac: accelerations; mtc: minimum toe clearance; si: stride intervals; f: forces.

**Ageing**		
*Metric:*	*Main trend:*	*Exceptions:*
Entropy	Increased entropy [79]^*jap*^, [80], [45], [57], [81]^*ac*^	[84]^*mtc*^, [44], [83]^*ac*^
MLE	Reduced stability [112]^*jap*^, [113]^*si*^, [108], [109]^*ac*^	-
MFA	Reduced correlations [148], [155]^*si*^	[154]^*si*^
Irreversibility	-	-

**Parkinson’s Disease**		
*Metric:*	*Main trend:*	*Exceptions:*
Entropy	Increased entropy [53], [86], [87], [90], [66]^*si*^, [88], [46], [47]^*f*^, [89]^*ac*^	[85]^*si*^
MLE	-	-
MFA	Reduced correlations [156], [144], [157]^*si*^, [95]^*f*^. Multi-fractal scaling [162], [168]^*si*^	[164], [165]^*si*^
Irreversibility	-	-

**Huntington’s Disease**		
*Metric:*	*Main trend:*	*Exceptions:*
Entropy	Increased entropy [85], [53], [86], [91], [90]^*si*^, [46]^*f*^	[51]^*f*^
MLE	-	-
MFA	Reduced correlations [148]^*si*^. Multi-fractal scaling [162]^*si*^	[164], [165]^*si*^
Irreversibility	-	-

**Alzheimer’s Disease**		
*Metric:*	*Main trend:*	*Exceptions:*
Entropy	Increased entropy [99]^*jap*^	-
MLE	-	-
MFA	-	-
Irreversibility	Mixed [99]^*jap*^	-

**Amyotrophic Lateral Sclerosis**		
*Metric:*	*Main trend:*	*Exceptions:*
Entropy	Increased entropy [53], [86]^*si*^, [51]^*f*^	[85]^*si*^
MLE	-	-
MFA	Reduced correlations [156]^*si*^. Multi-fractal scaling [163]^*si*^	[164], [165]^*si*^
Irreversibility	-	-

The attentive reader will already have identified some common patterns: pathologies usually increase entropy, and reduce stability and correlations. This is not completely surprising, as one may expect a weaker control by the central nervous system, and hence a less controlled gait. There are nevertheless exceptions, which point towards a more complex scenario. Any metric showing a more controlled system could be pointing at the deployment of adaptive mechanisms in the brain (while this is not too lesioned), to create a successful cinematic and kinetic configuration maintaining a functional walk. However, this control will fail as soon as the adaptive mechanisms fail, leading to a completely uncontrolled system. This is known to happen in motor neuron degenerative diseases, such as Amyotrophic Lateral Sclerosis and Multiple Sclerosis. Depending on the evolution of the desease, one can thus find a wide spectrum, from lower to higher entropy, when comparing against healthy subjects. Lesions in the basal ganglia may start with some increase in control at the debut of disease; yet, as basal ganglia are a very important part of the adaptive mechanism itself, the progression of the lesion will result in an intensifying lack of control.

Results here reported also highlights some challenges that will have to be overcome, in order to ensure that IGA will have a prominent role in clinical practice.

First of all, observing differences between e.g. control subjects and patients is not necessarily tantamount to obtaining useful clinical knowledge and tools. On the one hand, as the described metrics are related with the output of the motor system, their changes cannot directly be interpreted as causal mechanisms. Abnormalities in motor output can emerge as secondary adaptations of the system, requiring additional and tailored experiments to confirm any causal hypothesis. On the other hand, while most research works have focused on the statistical significance of results, only few have used such differences to build classification models [62], [86], [169], [95], [64], [46], [90], [66]. The importance of having such models, and specifically explainable ones, is nevertheless obvious, and is especially relevant in the case of pathologies for which early diagnosis is hindered by a lack of reliable and affordable early biomarkers - e.g. Parkinson’s and Alzheimer’s diseases.

Secondly, as illustrated in Table 3, many contradictory results have been observed for multiple pathologies, or even for control subjects in comparable conditions. On one hand, this may point towards an evolution of the underlying control strategy by part of the central nervous system; something that can only be confirmed through longitudinal studies. Yet, and on the other hand, this may also be the spurious result of using different techniques to record gait; of heterogeneous (and not easily comparable) data recording procedures and conditions; and of the use of different variants of the four metrics, of different algorithms to calculate them, or of different parameters. In spite of some efforts in the Literature [107], [68], [48], [35], [61], [67], [50], [69], [70], [67], [188], more comparative studies are needed to clarify which metrics, algorithms and parameter values ought to be used, possibly by relying on numerical models of gait [145], [146], [110].

Thirdly, one must acknowledge that creating bridges between different scientific fields is challenging at best, as concepts and ideas that are standard in one of them may be hard to grasp in another one. The case here reviewed is not exception, resulting in some widespread technical and conceptual fallacies. To illustrate, different metrics have different requirements in terms of minimum time series length - some very broad guidelines are included in Table 2. Yet, the Hurst exponent has been estimated on time series as short as 20 values [148], something that is known to lead to overestimations; interestingly, results were then confirmed with longer time series [162]. We have also found many examples of research works claiming that the complexity of the time series was calculated, for then using entropy metrics [29], [75], [68], [189], [26], [49], [71], [57], [50], [61], [190], [90]. While entropy and complexity are undoubtedly related concepts, they are not interchangeable, as well known in statistical physics [191], [192], [193]. This highlights the importance of relying on mixed teams, in which people with different background (medical on one hand, physics on the other) can interact.

As a final point, Table 3 (or even the length of the different sections) suggests that the four concepts here analysed, while being complementary in nature, have not equally been considered. Specifically, the use of entropy and MFA can be considered as widespread in gait analysis; but only two research works have focused on the irreversibility of gait time series. This may be the results of several factors. First of all, computational approaches to irreversibility are relatively recent, with the first metric being proposed by Yves Pomeau in 1982 [194] - entropy, in contrast, is a concept known in information theory since 1948 [30]. Additionally, while many more metrics have been proposed in the last decade, choosing the best one for a given real-world problem is not trivial [188]; the computational cost is usually much higher, when compared to entropy metrics [188]; and the understanding of the theoretical meaning of irreversibility is challenging.

In spite of these challenges ahead, it is clear that the instrumental analysis of gait can strongly benefit from statistical physics concepts; and that existing studies have helped in understanding the mechanisms behind some major pathologies and how they affect gait, from Parkinson’s to Alzheimer’s. It would be far from surprising to see an increase in the number of published papers on this topic; and even the adoption of those metrics in a clinical context in the near future.

## CRediT authorship contribution statement

**Massimiliano Zanin:** Conceptualization, Investigation, Writing - original draft, Writing - review & editing, Supervision. **Felipe Olivares:** Conceptualization, Investigation, Writing - original draft, Writing - review & editing. **Irene Pulido-Valdeolivas:** Conceptualization, Investigation, Writing - original draft, Writing - review & editing. **Estrella Rausell:** Conceptualization, Investigation, Writing - original draft, Writing - review & editing. **David Gomez-Andres:** Conceptualization, Investigation, Writing - original draft, Writing - review & editing.

## Declaration of Competing Interest

Irene Pulido-Valdeolivas has received travel reimbursement from Roche Spain, Novartis and Genzyme-Sanofi, and she is a founder and holds stock in Aura Robotics SL. She is an employee at UCB Pharma since July 2020 and all the work in this paper is based on her previous work at Universidad Autónoma de Madrid.

David Gómez Andrés has received honoraria as an advisor from Biogen and Lupin Neuroscience and as a speaker from Biogen, PTC, and Shire. He has also received travel reimbursement from Roche, PTC, Shire and Laboratorios Rubio. He was founder and holds stock in Aura Robotics SL. He is/was an investigator in clinical trials founded by Pfizer, Biogen, Avexis, Roche, Fibrogen and Santhera.
